# First Metatarsophalangeal Joint Arthrodesis in Hallux Valgus Versus Hallux Rigidus Using Cup and Cone Preparation Compression Screw and Dorsal Plate Fixation

**DOI:** 10.7759/cureus.1786

**Published:** 2017-10-19

**Authors:** Calvin Chien, Terrence Alfred, Richard Freihaut, Sabrina Pit

**Affiliations:** 1 Orthopaedics, NSW Health; 2 Orthopaedics, Lismore Base Hospital; 3 School of Medicine, University Centre for Rural Health, Western Sydney University, Lismore

**Keywords:** hallux valgus, hallux rigidus, first metatarsophalangeal joint, arthrodesis

## Abstract

Various techniques have been described for first metatarsophalangeal (MTP) joint arthrodesis. The purpose of this study was to determine if cup and cone preparation by a single surgeon with an interfragmentary screw and dorsal plate fixation provides a comparable union rate in hallux valgus versus hallux rigidus. Our study included all patients who underwent first MTP joint fusions using cup and cone preparation with an interfragmentary compression screw and dorsal plate fixation from 2010 to 2015. We compared union rates in 65 patients with hallux rigidus with 47 who had hallux valgus. One of 65 hallux rigidus cases developed non-union and underwent revision surgery. One of 47 patients in the hallux valgus group developed a painless non-union. All other patients achieved union based on post operative radiographs. Our rate of painful non-union was 1.5% for hallux rigidus and 0% for hallux valgus, which is lower than recent published literature of 7% for hallux valgus and 3.7% for hallux rigidus. We found no difference between the two groups suggesting this method may provide stronger fixation and may be preferable when dealing with hallux valgus. First metatarsophalangeal joint fusion in patients with severe hallux valgus and hallux rigidus, using spherical reamers, compression screw and dorsal plate fixation is equally successful at achieving clinical and radiographic fusion in both hallux valgus and hallux rigidus.

## Introduction

Various techniques have been described for first metatarsophalangeal (MTP) joint arthrodesis. In preparing the joint surfaces biplanar saw cuts, use of rongeurs and specific reamers have all been utilized. Furthermore, the fixation methods include Kirschner wires, interfragmentary screws, a dorsal plate or a combination. All techniques give satisfactory results in the treatment for severe hallux valgus as well as hallux rigidus [1-4]. A review of the literature showed that nonunion following MTP joint arthrodesis for severe hallux valgus averaged 7%, almost double for hallux rigidus averaging 3.7% [5]. In addition, a recent single surgeon study had concluded that hallux valgus was associated with higher non-union rates [5]. However, biplanar cuts were used in all cases and either crossed screw fixation in the majority or a dorsal plate with or without a compression screw. It may be that the hallux valgus group needs a stronger construct to achieve comparable union rates to the hallux rigidus group [6].

The purpose of this study was to determine if cup and cone preparation by a single surgeon with an interfragmentary screw and dorsal plate fixation provides a comparable union rate in hallux valgus versus hallux rigidus.

## Materials and methods

Data collection

From a database of cases performed by the senior author, data from all first MTP joint fusions using cup and cone preparation with an interfragmentary compression screw and dorsal plate fixation from 2010 to 2015 was obtained. Clinical and radiographic records were collected and were then divided according to pathology.The Picture Archiving and Communication System (PACS) was used to collect preoperative and postoperative weight-bearing radiographic data. The intermetatarsal angle (IMA) and hallux valgus angle (HVA) were recorded. All the cases were followed up until satisfactory radiographic union (bone bridging of three out of four cortices) had been achieved or the fusion was asymptomatic. This ranged from six to 26 weeks. Furthermore, where possible, patients were clinically followed up at the time of data collection to determine if they had had revision surgery elsewhere other than hardware removal.

Sample

There was a total of 135 first MTP joint arthrodeses performed within the time frame. Twenty-three cases were excluded. Three had hallux varus, ten had inflammatory arthritis, five were salvage procedures that had recurrent hallux valgus and five were previously failed first MTP joint arthrodeses that had revisions. This left 112 cases in the study. Sixty-five hallux ridigus and 47 hallux valgus that were primarily fused.

Operative technique

**Figure 1:**
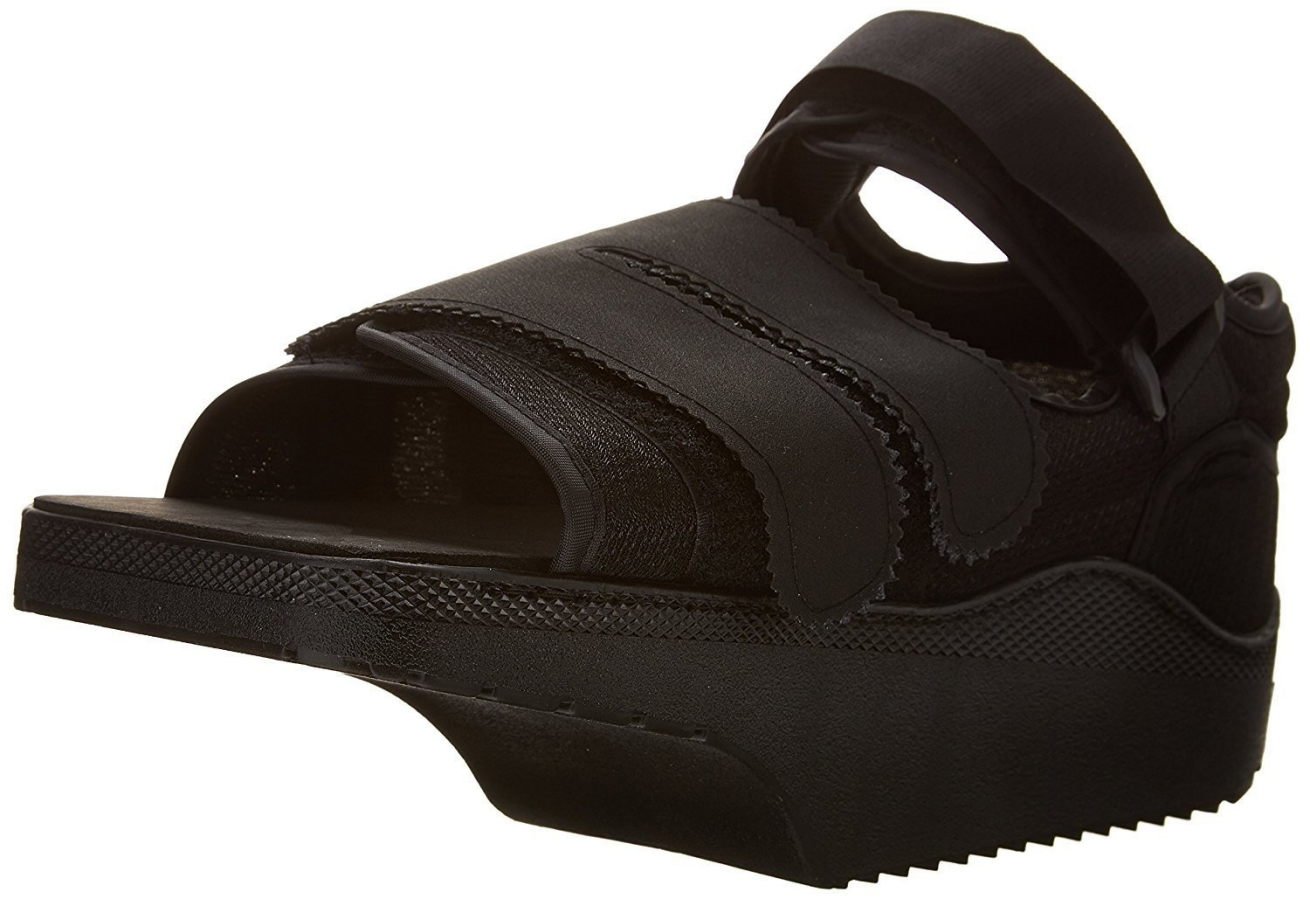
Heel wedged velcro shoe

**Figure 2:**
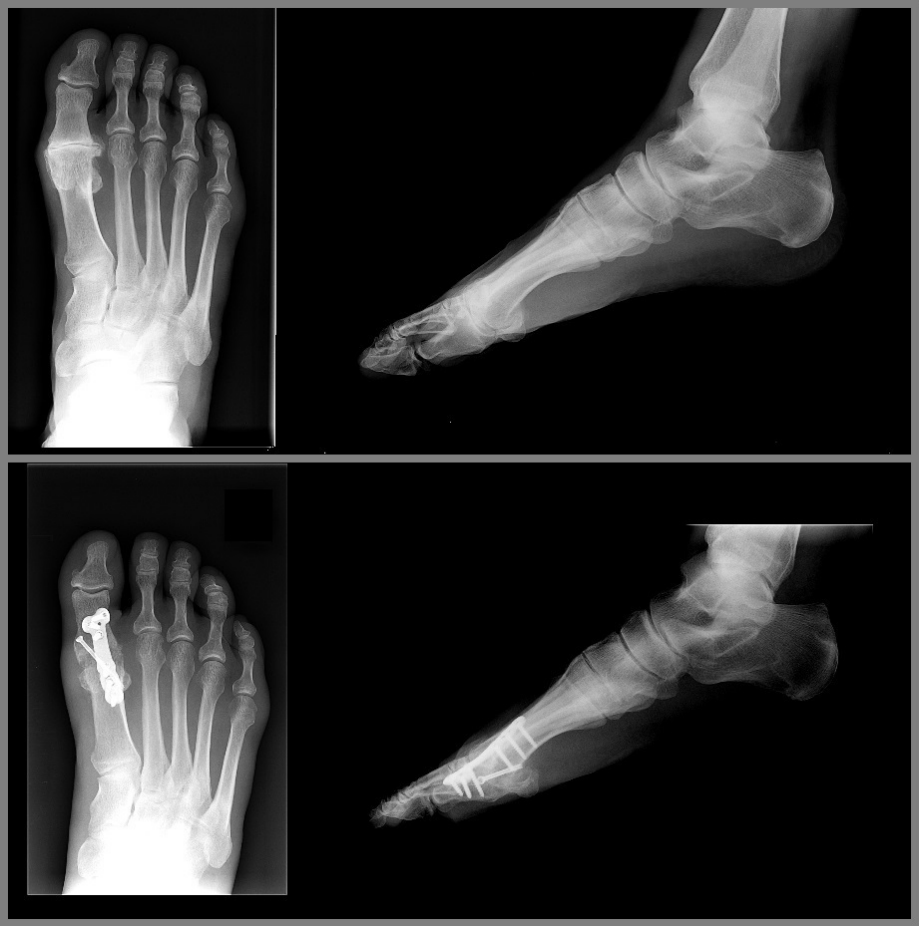
Hallux rigidus pre- and postoperative

**Figure 3:**
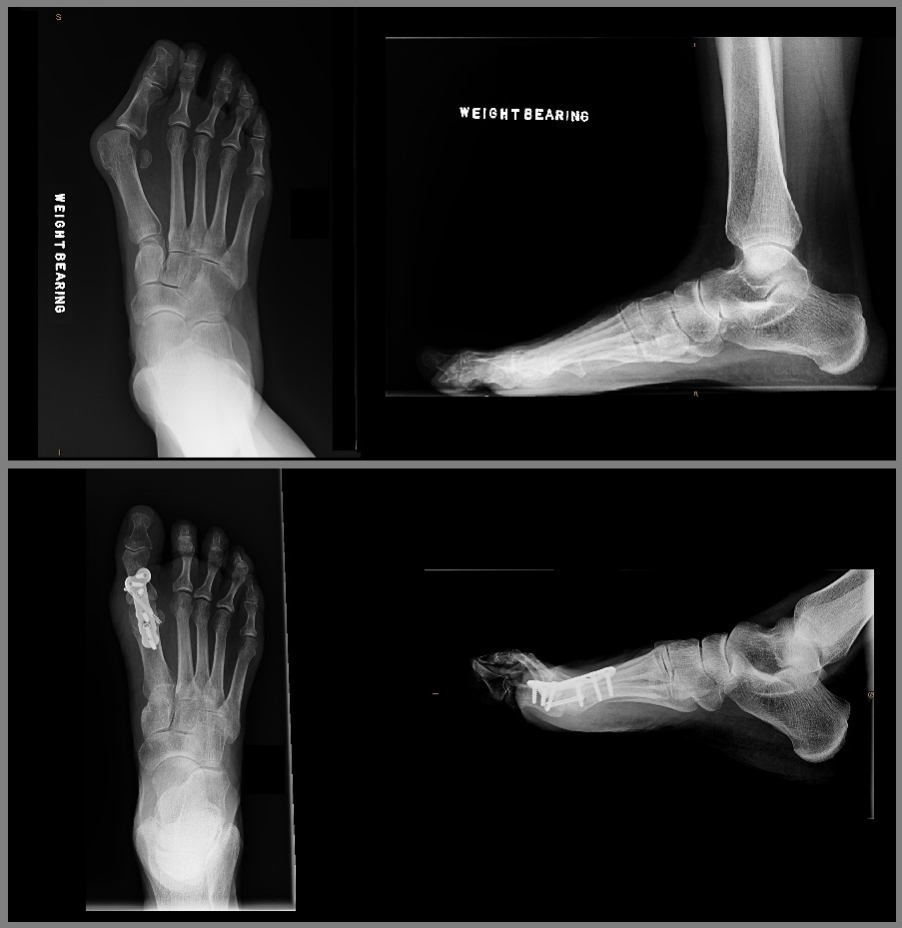
Hallux valgus pre- and postoperative

With the patient supine and a tourniquet inflated, a longitudinal dorsal incision was used to access the MTP joint. A capsulotomy was performed at the medial edge of the extensor hallucis longus tendon and the joint was subluxated. Osteophytes were excised and specialized reamers were then used to prepare the joint surfaces, the cup being distal and cone proximal, taking care to avoid excessive shortening. This then allowed the joint to be easily positioned with a hallux valgus angle of 0-10 degrees. The appropriate amount of dorsiflexion was achieved to allow the toe to sit 1 cm off the “floor” with the interphalangeal joint dorsiflexed and to touch the “floor” with the interphalangeal joint plantar flexed. A 3.0 mm cannulated lag screw was initially used to provide compression. A dorsal locking plate was then used in all cases for added stability. The pre-contoured plate was adjusted if necessary to increase or decrease the amount of dorsiflexion to fit the bone. It was fixed with two non-locking screws to compress the plate to the bone and the remaining holes were filled with locking screws. The position was checked with a mobile image intensifier. The capsule was then repaired and the skin closed with non-absorbable horizontal mattress sutures. A compression bandage was applied. A plaster cast was not used. The patients were kept heel weight-bearing for six weeks in a heel wedged velcro shoe (Figure 1). Initial follow up was at two weeks for removal of sutures. At six weeks a radiographic examination was performed to assess bone bridging. Union was defined by bone bridging in three out of four cortices on plain X-ray. If satisfactory, the patient was allowed to fully weight bear otherwise further follow up was arranged. Failure was defined as needing revision surgery for a painful non-union (Figures 2, 3).

Outcome measure

The main outcome measure was painful non-union.

Statistical analyses

SAS 9.4 (SAS Institute Inc., Cary, NC) was used to analyze the data. The Chi-square and Fisher Exact tests were used to compare binary outcome measures. The Wilcoxon Ranksum test was used to compare age and follow up length, in weeks, between the two groups.

## Results

Demographics between the two groups are shown in Table 1. The number of female cases was 80 and male cases 32. Mean and median age (at the time of operation) was 65.5 years (interquartile range: 60-73; minimum: 22; maximum 85 years). There was a statistically significant difference between hallux rigidus and hallux valgus with respect to age (P<0.0001). Patients with hallux valgus presented for surgery later in life than those with hallux rigidus. There was, however, no difference between the groups with respect to gender and length of follow up. The pre-operative hallux valgus angle and intermetatarsal angle values for the two groups are shown in Table 2.

**Table 1:** Demographics and union rate with respect to pathology (N=112) *Chi-square test, **Wilcoxon Rank Sums test, *** Interquartile range, **** Fisher Exact Test

	Hallux Rigidus (n=65)	Hallux Valgus (n=47)	P-value
Gender	Number (%)	Number (%)	
Female	45 (69%)	35 (75%)	0.54*
Male	20 (31%)	12 (25%)	0.54*
Age	Median*** (range)	Median*** (range)	
	61 years (57 to 69)	72 years (65 to 77)	<.0001**
Follow up (range)	6 weeks (6 to 14)	6 weeks (6 to 14)	0.91**
Painful Non-Union	Number (%)	Number (%)	
Yes	1 (1.5%)	0 (0%)	1.00****
No	64 (98.5%)	47 (100%)	1.00****

**Table 2:** Intermetatarsal angle and hallux valgus angle with respect to pathology

	Intermetatarsal Angle		Hallux Valgus Angle	
	Average	Range	Average	Range
Hallux Rigidus	9	5-16	12	3-24
Hallux Valgus	15	6-23	39	20-55

Union rates

Out of the 65 hallux rigidus cases, one developed non-union and underwent revision surgery (Table 1). All 47 hallux valgus cases developed bone bridging or painless non-union at follow up and have not had revision surgery. Bone bridging occurred in 46 out of 47 hallux valgus joints with one patient developing painless non-union. There was no association between union rate and pathology (P=1.00).

## Discussion

The rate of painful non-union was 1.5% for hallux rigidus and 0% for hallux valgus. This is slightly lower than recent published literature of 7% for hallux valgus and 3.7% for hallux rigidus [5], however fusion success rates have been reported in the literature at 80% - 100% [4]. Our results were comparable to a recent study by Kumar, et al., who used a similar operative technique for 46 patients with hallux valgus or hallux rigidus, and achieved a union rate of 98% [4]. Ellington, et al., in their 2010 paper, however, achieved fusion in only 86.2% of their 87 patients having a primary arthrodesis using the same technique [7]. Their paper however included patients with rheumatoid arthritis, and showed that these patients had a lower union rate of only 77.1%. Ellington, et al., also included patients undergoing revision arthrodesis, achieving a 95% union rate in 20 patients. Our paper excluded patients with inflammatory arthropathies and those undergoing revision and salvage surgery. Korim and Allen performed a similar case series to ours in 2010 using flat cuts and cross screw fixation [5]. Their non-union rates were 6% in the hallux valgus group, and 0% in the hallux rigidus group. They hypothesised that the poorer bone quality and deforming forces in hallux valgus may compromise fixation using flat bone cuts and crossed screw technique. The same authors also trialled fusion using memory staples, however abandoned that fixation method because of unacceptably high non union rates. Using cup and cone reaming with compression screw and dorsal plate fixation we found no difference between the two groups suggesting this method may provide stronger fixation and may be preferable when dealing with hallux valgus.

Strengths of this study include that this was a single surgeon series, with a consistent technique of joint preparation and fixation over six years. In addition, both groups had similar numbers of patients with similar demographics in this relatively large case series. The study’s main weakness is its retrospective design with too few numbers to achieve significance. The patient follow-up period was relatively short in some instances, but only when union was identified radiographically, indicating a successful surgical outcome. Our study also lacked patient-reported outcome scores.

## Conclusions

First metatarsophalangeal joint arthrodesis in patients with severe hallux valgus and hallux rigidus, using spherical reamers, compression screw and dorsal plate fixation is equally successful at achieving clinical and radiographic fusion in both hallux valgus and hallux rigidus. Crossed screw fixation may be sufficient when treating hallux rigidus, however, the deforming forces and poor bone quality of severe hallux valgus may necessitate the need for a stronger construct. Future randomized and prospective studies are required to better assess arthrodesis methods and results in patients with different pathologies.

## References

[1] Coughlin MJ, Grebing BR, Jones CP (2005). Arthrodesis of the first metatarsophalangeal joint for idiopathic hallux valgus: intermediate results. Foot Ankle Int.

[2] Flavin R, Stephens MM (2004). Arthrodesis of the first metatarsophalangeal joint using a dorsal titanium contoured plate. Foot Ankle Int.

[3] Hunt KJ, Ellington JK, Anderson RB, Cohen BE, Davis WH, Jones CP (2011). Locked versus nonlocked plate fixation for hallux MTP arthrodesis. Foot Ankle Int.

[4] Kumar S, Pradhan R, Rosenfeld PF (2010). First metatarsophalangeal arthrodesis using a dorsal plate and a compression screw. Foot Ankle Int.

[5] Korim MT, Allen PE (2015). Effect of pathology on union of first metatarsophalangeal joint arthrodesis. Foot Ankle Int.

[6] Buranosky DJ, Taylor DT, Sage RA (2001). First metatarsophalangeal joint arthrodesis: quantitative mechanical testing of six-hole dorsal plate versus crossed screw fixation in cadaveric specimens. J FootAnkle Surg.

[7] Ellington JK, Jones CP, Cohen BE (2010). Review of 107 hallux MTP joint arthrodesis using dome-shaped reamers and a stainless-steel dorsal plate. Foot Ankle Int.

